# Clinical Characteristics and the Prognostic Factors of Acute Peripheral Facial Palsy in Children

**DOI:** 10.3390/medicina61101790

**Published:** 2025-10-03

**Authors:** Young-Soo Chang, Su Jeong You

**Affiliations:** 1 Department of Otorhinolaryngology-Head and Neck Surgery, College of Medicine, Inje University, Sanggye Paik Hospital, Seoul 01757, Republic of Korea; yschang83@gmail.com; 2 Department of Pediatrics, College of Medicine, Inje University, Sanggye Paik Hospital, Seoul 01757, Republic of Korea

**Keywords:** acute peripheral facial palsy, child, outcome, steroid

## Abstract

*Background and Objectives*: This study aimed to investigate the clinical characteristics of children with acute peripheral facial palsy (PFP) in a single medical center. *Materials and Methods*: We conducted a retrospective analysis of children under 19 years of age treated for acute PFP between January 2009 and July 2023. A total of 109 patients were enrolled, and 7 patients showed recurrence. Of all the patients included, only 68 patients followed up for over 1 month (the follow-up cohort), in whom clinical outcomes were analyzed. The recovery period was calculated as the duration between the first day of facial palsy development and the last follow-up day on which the patient achieved a House–Brackmann (H–B) grade of I or II. Patients were categorized into two groups depending on the initial severity of their facial palsy using the H–B grade: “incomplete palsy”, defined as H–B grade ≤III; and “complete palsy”, defined as H–B grade ≥IV. *Results*: When comparing the group of patients which had recurrence (n = 7) and the group with no recurrence (n = 91), the age in the group with recurrence was younger (79.1 ± 29.9 vs. 143.8 ± 52.5, months, *p* = 0.001, Mann–Whitney U test). In the follow-up cohort, four (5.9%) patients continued to have mild facial palsy (H–B grade II). Age, the time lag between the onset of palsy and treatment initiation, the type of etiology and the use of antiviral agents were not associated with the recovery period. The initial severity of facial palsy was significantly associated with the recovery period (incomplete palsy group 26.7 ± 18.4 days, complete palsy group 75.1 ± 96.0 days, unstandardized regression coefficient = 50.5, *p* = 0.001). *Conclusions*: The outcome of acute PFP in children showed a good prognosis. The recurrence of PFP was observed in younger patients. An initial severity of “complete palsy” entailed a significantly longer recovery period.

## 1. Introduction

Acute peripheral facial palsy (PFP) is a frequent and urgent condition in pediatric care. Although the annual incidence of PFP is more common in the general population (13–28 per 100,000 based on population-based studies), it still occurs at a considerable incidence in children (5–21 per 100,000 children annually) [1,2]. The actual incidence rate of each etiology in pediatric PFP varies across studies. Generally, the most common etiologies of PFP include Bell’s palsy, accounting for 60 to 80% of cases, followed by infectious diseases (4 to 37% of cases), trauma, cancer, and congenital abnormalities.

The prognosis of PFP in children is generally excellent. For the most common cause, Bell’s palsy, complete functional recovery usually occurs within two weeks of symptom onset and it has a spontaneous recovery rate of up to 97% in children [2]. The prognosis of PFP with other etiologies may differ depending on the treatment outcome of underlying diseases [3,4]. Although the prognosis and treatment of PFP in children depends on its etiologies and are known to be generally excellent, there is little evidence concerning the proper management of PFP in children. Two systematic reviews found that the most prescribed medication, corticosteroids, did not have sufficient evidence for it to be a treatment recommendation for pediatric Bell’s palsy [5,6]. The addition of antiviral therapy is also still controversial even in the adult population [7]. Considering that incomplete recovery from impaired facial movements affects the quality of life, coupled with the lack of documentation of the natural course of pediatric PFP, it is important to elucidate the clinical characteristics and the prognostic factors of PFP in children to set a timely intervention window and treatment recommendations.

In the present study, we aimed to investigate clinical characteristics and prognostic factors to predict the outcome of PFP in children.

## 2. Materials and Methods

### 2.1. Patient Population

We conducted a retrospective analysis of children under 19 years of age treated for PFP between January 2009 and July 2023. The criteria for inclusion were a diagnosis of acute peripheral PFP and that all the patients were initially treated for PFP in a single medical center. Patients with facial palsy after birth (n = 7), patients with head trauma (n = 3), and a patient with bone involvement of leukemia (n = 1) were excluded from the analysis. Of all the patients included, seven were treated for recurrent PFP. Sixty-eight patients followed up for over one month (the follow-up cohort), in whom clinical outcomes were analyzed.

This study was approved by the Institutional Review Board (IRB No. 2024-06-009). The IRB confirmation allowed informed consent to be waived because this study was a retrospective study on existing data that was conducted in accordance with general examination guidelines and was analyzed anonymously.

### 2.2. Methods

Information including age at onset, sex, history of infection and vaccination, side and severity of facial palsy, laboratory findings, magnetic resonance imaging (MRI) findings, treatments, timing of intervention, and outcomes were collected. All patients were examined by an otolaryngologist or a pediatrician during the initial workup. The possible etiologies of all included patients were analyzed.

All patients received oral prednisolone (1–2 mg/kg/day up to 60 mg/day, typically for 5–7 days followed by a tapering schedule over the next 5–7 days). Thirty-eight patients received Acyclovir as an antiviral agent (15–20 mg/kg/dose, administered orally every 8 h for 5–7 days). MRI was not routinely performed in typical cases of PFP. Clinical indications for MRI included atypical presentation, suspicion of a central nervous system cause, or the presence of red flag signs such as hearing loss or associated neurologic abnormalities. The decision to perform imaging was made at the discretion of the treating physician, and MRI was also recommended in some recurrent cases or in children younger than five years of age.

The House–Brackmann (H–B) grading system was used to classify the severity of facial palsy. The grading was performed by the examining otolaryngologist or pediatrician during the clinical workup. When patients were jointly evaluated in both specialties, the grading was assessed by both specialists, whereas in cases seen by a single department, the grading was performed by the attending specialist. Patients were categorized into two groups depending on the initial severity of their facial palsy as measured using the H–B system: “incomplete palsy”, defined as H–B grade ≤ III; and “complete palsy”, defined as H–B grade ≥IV. In the H–B grading system, grades I and II are normal to near-normal, whereas grades III or worse describe a status which has definite asymmetry. Therefore, a favorable outcome of PFP was set as H–B grades I or II in this study.

The recovery period was calculated as the duration between the first day of facial palsy development and the last follow-up day on which the patient achieved a House–Brackmann (H–B) grade of I or II. A multivariable linear regression analysis performed in the follow-up cohort to evaluate the possible factors associated with the recovery period, age, the time lag between palsy onset and treatment initiation, the type of etiology, the use of antiviral agents, and the initial severity of the facial palsy was adopted for analysis. In addition to the primary seven-day cut-off for treatment initiation, a sensitivity analysis using a 72 h threshold was also conducted.

### 2.3. Statistical Analysis

All data was expressed as mean ± standard deviation (SD). For skewed variables, median values with interquartile ranges (IQRs) were additionally reported. Fisher’s exact test was applied for categorical data, and the Mann–Whitney U test was used for continuous variables. Multivariable logistic regression analysis was performed to identify prognostic factors, with odds ratios (ORs) and 95% confidence intervals (CIs) reported. Explicit reference groups were defined for each categorical variable. To ensure the stability of the regression model, collinearity diagnostics were performed using the variance inflation factor (VIF), and residual analysis was conducted. A two-sided *p*-value of < 0.05 was considered statistically significant. All analyses were performed using SPSS software, version 20.0 (IBM Co., Armonk, NY, USA).

## 3. Results

**Figure 1:**
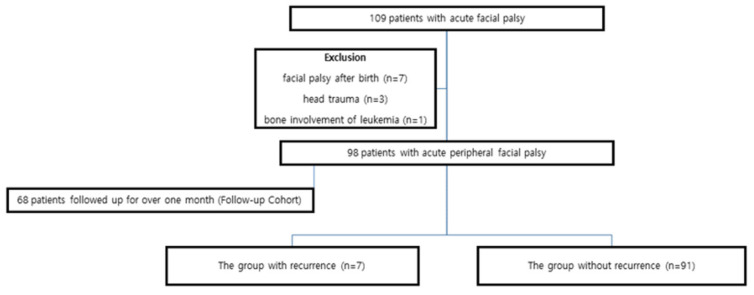
Study’s flow diagram of patients with acute facial palsy.

A total of 109 patients were diagnosed with acute PFP. Following the exclusion criteria, an analysis was performed on a total of 98 patients with acute peripheral PFP (Figure 1).

Demographic characteristics of the children according to the presence or absence of PFP recurrence are shown in Table 1. Of the 98 patients, 7 patients were treated for recurrent PFP (incidence rate 7.14%). Comparing average ages at initial PFP presentation, the group without recurrence experienced PFP at the age of 143.8 ± 52.5 months and the group with recurrence experienced PFP at the age of 79.1 ± 29.9 months. The result shows a significant difference (*p* = 0.001, Mann–Whitney U test). Twenty-one patients (21.4%) underwent MRI studies. Among them, two patients exhibited facial nerve enhancement, while the remaining patients showed no specific findings.

The characteristics of each patient in the group with recurrent PFP (n = 7) are presented in Table 2. Four patients suffered acute peripheral PFP twice and other patients suffered either three or four events of acute peripheral PFP. Three patients suffered acute peripheral PFP on different sides of the face (Patient number one, two, and four). All of them received steroid treatment and they showed favorable outcomes at every event regardless of the initial severity of their PFP.

### Analysis of the Follow-Up Cohort

The mean age at onset (sixty-eight patients followed up for over one month) was 131.9 ± 53.5 months (±SD), ranging from 12 months to 227 months (Table 3). Sixty patients were diagnosed as having Bell’s palsy. Six patients (three with otitis media and three with Ramsay Hunt syndrome) were associated with definite infectious causes. Two patients developed PFP following vaccination (one for influenza 7 days post-vaccination, and one for Pfizer COVID-19 4 days post-vaccination), which was documented as a temporal association rather than evidence of a causal relationship. The number of patients with incomplete palsy was 50 (73.5%) and the number with complete palsy was 18 (26.5%). All of them received oral prednisolone (1–2 mg/kg/day up to 60 mg/day) and the mean time until steroid initiation was 2.8 ± 3.1 (SD) days, ranging from 0 days to 16 days. Thirty-eight patients received antiviral agents as the initial treatment (15–20 mg/kg/dose, every 8 h). All of the follow-up cohort patients showed favorable outcomes in their treatment for PFP on their last visit.

The results of the multivariable logistic regression analysis are presented in Table 4 (R^2^ = 0.24). The variables analyzed included age (reference group: < 6 years), the time lag between palsy onset and treatment initiation (reference group: < 7 days), the type of etiology (reference group: Bell’s palsy), the use of antiviral agents (reference group: YES), and the initial severity of facial palsy (reference group: incomplete palsy). Age, the time lag between palsy onset and treatment initiation, the etiology, and the use of antiviral agents were not associated with the recovery period. However, patients with initially complete facial palsy had significantly higher odds of incomplete recovery compared to those with incomplete facial palsy.

## 4. Discussion

In our study, we reported several unique clinical characteristics among pediatric acute PFP patients based on an analysis of patient data collected over a 15-year period at a single institution.

First, we identified seven patients (7/98, 7.14%) who experienced recurrent PFP, which is consistent with previous studies that reported a similar incidence of approximately 6% in children [8]. These patients exhibited early onset facial palsy, with recurrences on either the same or contralateral side. Notably, all cases of recurrent PFP in our study resulted in favorable outcomes, in contrast to the findings of Eidlitz-Markus et al. who reported a complete clinical recovery rate of only 70% for recurrent PFP [8]. This discrepancy may be associated with the initial age of onset; Eidlitz-Markus et al. observed recurrent PFP in children approximately two years older than that of our patients when their patients visited the clinic with Bell’s palsy. Our patients were significantly younger at the time of initial onset compared to those with non-recurrent PFP (79.1 ± 29.9 months vs. 143.8 ± 52.5 months, Mann–Whitney U test, *p* = 0.001). Our findings suggest that younger children may be more prone to recurrent PFP, potentially due to developmental factors or differences in immune response. The immaturity of the immune system in younger children could contribute to their increased vulnerability to recurrent episodes [9,10]. Additionally, incomplete myelination of the facial nerve in early childhood may play a role, as the developing nervous system, including ongoing myelination, could influence both the susceptibility to nerve injury and the capacity for recovery [11]. These factors indicate the need for close monitoring and, potentially, modified therapeutic approaches in younger children to prevent recurrences and ensure optimal recovery as myelination progresses. However, despite these observations, few studies have focused on recurrent PFP in children. Further research is essential to better understand the clinical characteristics and prognostic factors associated with favorable outcomes in pediatric patients with recurrent PFP.

Second, in the analysis of patients followed up for over one month (n = 68), we demonstrated that a diagnosis of “complete PFP” in their first visit was significantly associated with a longer recovery period. It is known that patients with partial paralysis (incomplete PFP) have favorable prognoses [12,13,14]. A recent retrospective cohort study on pediatric Bell’s palsy patients found that the initial severity of facial paralysis was the most significant prognostic factor for complete recovery. Specifically, a lower initial H–B grade (II–IV) was associated with a more favorable outcome at six months (OR: 3.86; 95% CI: 1.27–11.70; *p* < 0.05). This finding aligns with our results, which also demonstrated that the initial severity of facial palsy was significantly associated with the recovery period in children with acute peripheral facial palsy [13]. In addition, our results suggested that patients with complete PFP during their first visit require an extended period of follow-up compared to the patients with incomplete PFP. With the extended period of follow-up, all the patients with complete PFP recovered with a favorable outcome of H–B grade II or lower, which has been reported in numerous studies to be determined by normal function in daily life [15,16]. Prognostic factors in adults with Bell’s palsy have been well defined, with favorable outcomes reported in 80–94% of adult patients treated either with or without combination antiviral therapy [17,18,19]. In addition, previous studies have shown that pediatric patients with Bell’s palsy had a better prognosis than adult patients, suggesting that younger age was associated with a positive prognosis [17,20]. Our study observed that all patients who underwent long-term follow-up had favorable outcomes. This finding not only supports previous findings but also provides valuable information that can aid in counseling pediatric patients with complete PFP of various etiologies.

Although the role of steroid treatment for facial palsy in children is inconclusive, we prescribed corticosteroids for all the included patients [5]. A previous study has reported that childhood Bell’s palsy showed a good prognosis both with or without corticosteroid treatment, and the authors suggested that steroid therapy in childhood Bell’s palsy may not significantly improve outcomes. However, another study suggested that using prednisolone to treat Bell’s palsy in children is likely cost-effective compared with a placebo over a six-month period in children aged 12 to 17 years in terms of children’s quality of life and quality-adjusted life-years, which are increasingly valued by clinicians in practice [21]. Improving children’s quality of life and quality-adjusted life-years is associated with earlier recovery from PFP, which is demonstrated by the results of a randomized control trial with 42 children to evaluate the effects of methylprednisolone; it showed that children receiving methylprednisolone recovered earlier [22]. In addition, the time at which steroid initiation should begin is still controversial. Although one study recommended that steroid treatment start within 72 h of diagnosis, most previous studies have reported that the time of steroid initiation was not a prognostic factor in pediatric patients with PFP [14,23,24]. In our study, all pediatric patients were treated with steroids within two weeks of the onset of PFP, and the time lag was not associated with recovery. A sensitivity analysis using a 72 h cutoff for steroid initiation also showed no significant association with the outcome. However, we observed that all the included patients recovered with a favorable outcome regardless of recurring or initial severity. Therefore, further research on the significance and necessity of steroid treatment in pediatric PFP is needed.

Among the patients not included in this study’s analysis, one patient without any history of trauma or inflammation who presented with PFP was ultimately diagnosed with leukemia through a blood test. This case underscores the need for a thorough diagnostic evaluation in pediatric PFP, as the common practice of diagnosing unilateral PFP as Bell’s palsy and initiating steroid therapy without further investigation may overlook underlying systemic conditions. As such, the differential diagnosis should remain broad. While Bell’s palsy is the most frequent cause, other etiologies such as infection, trauma, and, more rarely, tumors like facial nerve schwannoma must be considered, especially in cases with atypical presentations or a lack of improvement [25,26]. A recent large-scale retrospective cohort study by Walsh et al. (2021) examined the risk of malignancy following Bell’s palsy diagnosis in children presenting in the emergency department [27]. They found a small but clinically significant rate (0.33%) of new onset oncologic diagnoses within 60 days after Bell’s palsy diagnosis, with a higher incidence in children under five years old. The Walsh study underscores the importance of comprehensive evaluation in pediatric facial nerve palsy cases, aligning with our findings on the need for careful assessment of underlying conditions in children presenting with facial palsy. A previous review suggested several ‘red flag’ signs of pediatric PFP including hearing loss, associated neurologic abnormalities, involvement of a single branch of the facial nerve, progression of paralysis beyond three weeks, recurrent PFP, lymphadenopathy, bleeding manifestations, hypertension, and parotid mass [12]. The presence of any of these signs should prompt further investigation, including a detailed clinical examination and basic laboratory tests such as a complete blood count. This case of leukemia presenting as PFP illustrates the potential for systemic conditions to be initially misdiagnosed and highlights the need for increased awareness among clinicians. Given the risk of misdiagnosing serious conditions, we propose a stepwise diagnostic approach for pediatric PFP. Initial investigations should include a full clinical history, blood tests, and appropriate imaging in cases with red flag signs or atypical presentations. Such an approach may lead to the earlier detection of systemic diseases such as leukemia, thereby improving patient outcomes.

This study has several limitations. First, it is important to keep in mind that these results are based on a retrospective study conducted at a single institution. The study period also included the COVID-19 pandemic. Although we did not present detailed year-by-year case numbers in this manuscript, among the 98 patients included, there was no apparent clustering or unusual increase in pediatric facial palsy cases during 2020–2022 compared with other periods. Second, electromyography (EMG) studies and synkinesis assessments were not conducted in the patients. EMG provides valuable insights into clinically undetectable factors, such as motor unit potentials in paralyzed muscles (an important prognostic factor) and synkinetic activity, by placing a needle electrode in one muscle while instructing the patient to move another facial muscle [28,29]. Considering that 10–30% of idiopathic facial palsy cases develop synkinesis [30], performing a clinical assessment of synkinesis or EMG is important. However, due to the invasive nature of needle EMG and considering that pediatric patients may find the test difficult to tolerate, it was not conducted at the time. Therefore, when interpreting the results of this study, it should be noted that the evaluation of synkinesis and EMG assessment were not adequately conducted. Furthermore, an important methodological limitation is that the H–B grading was clinician-dependent and performed without any standardized inter-rater reliability assessment, which may have introduced variability in severity classification. Third, although all patients with recurrent facial palsy achieved complete recovery, MRI was performed in only about one-fifth of the total cohort and was selectively obtained in atypical, recurrent, or very young cases. This limited and non-standardized use of MRI introduced a potential selection bias and may have led to insufficient evaluation for other underlying conditions. However, our long-term follow-up data has produced valuable insights into the clinical characteristics of recurrent PFP patients and the prognostic factors of pediatric PFP. Future multicenter studies with larger sample sizes are warranted to validate our results and provide more robust evidence for guiding clinical practice. Furthermore, assessing synkinesis and conducting surface EMG evaluations would be valuable in investigating the potential role of steroid treatment in pediatric patients with PFP.

## 5. Conclusions

In conclusion, our study underscores the importance of comprehensive evaluation and tailored management strategies in pediatric PFP. A novel finding of our study is that recurrent PFP tends to occur in younger children and that complete palsy at initial presentation has been identified as a significant prognostic factor for incomplete recovery. By elucidating the clinical characteristics and prognostic factors influencing these outcomes, we aim to facilitate timely interventions and optimize treatment recommendations for pediatric patients with PFP. Further prospective studies with larger sample sizes are needed to validate these findings and explore tailored management strategies, particularly regarding treatment timing, recurrent PFP risk factors, and the role of imaging studies in pediatric cases.

## Figures and Tables

**Table 1:** Baseline characteristics according to the recurrence.

	Group Without Recurrence (n = 91)	Group with Recurrence (n = 7)	Comparison
Sex			
Male	44	4	*p* = 0.48
Female	47	3	chi-squared
Age (months)	143.8 ± 52.5	79.1 ± 29.9	*p* = 0.001 Mann–Whitney U test

**Table 2:** Characteristics of patients with recurrent facial nerve palsy.

	Age (Months)	Steroid Initiation (Days)	Recovery Period (Days)	Side	Initial Severity (H–B Grade)	MRI
Patient 1						
1st	107	1				
2nd	151	2	28	Lt	V	Facial nerve enhancement, Lt
3rd	239	3	6	Rt	II	
Patient 2						
1st	99	8		Rt	III	
2nd	199	6	14	Lt	III	
Patient 3						
1st	81	7	21	Rt	IV	
2nd	155	0	5	Rt	III	
Patient 4						
1st	105	7		Lt	III	
2nd	226	1	39	Rt	IV	
Patient 5						
1st	72	1	6	Lt	IV	
2nd	138	1	5	Lt	III	
Patient 6						
1st	21	0		Rt		
2nd	48	0	8	Rt		Facial nerve enhancement, Rt
3rd	125	2	6	Rt	III	Normal findings
Patient 7						
1st	69		14			Normal findings
2nd	95		14	Lt		
3rd	110	0	6	Lt	IV	
4th	176	1	11	Lt	III	Normal findings

SD, standard deviation; H–B grade, House–Brackmann grading

**Table 3:** Characteristics of patients followed up for over one month (the follow-up cohort).

	Patients Followed up for over One Month (n = 68)
Sex	
Male	34 (50%)
Female	34 (50%)
Age (months)	
Mean (± SD)	131.9 ± 53.5
Range	12–227
Etiology	
Bell’s palsy	60 (88.2%)
Otitis media	3 (4.4%)
Ramsay-Hunt syndrome	3 (4.4%)
Vaccination	2 (2.9%)
Steroid initiation (days)	
Mean (± SD)	2.8 ± 3.1
Range	0–16
Treatment	
Steroid	68 (100%)
Antiviral agents	38 (55.9%)
Recovery period (days)	
Mean (± SD)	39.5 ± 55.2
Median (IQR)	28 (21)
Range	6–365
Initial severity (H–B grade)	
II	11 (16.2%)
III	39 (57.4%)
IV	18 (26.5%)
Final outcome (H–B grade)	
I	64 (94.1%)
II	4 (5.9%)

SD, standard deviation; H–B grade, House–Brackmann grading.

**Table 4:** Prognostic factors for incomplete recovery in the follow-up cohort (n = 68) by logistic regression analysis.

Variable	Categories	Odds Ratio (OR)	95% CI	*p*-Value
Initial Severity	Complete palsy	5.83	1.48–22.97	0.01 *
	Incomplete palsy	Reference		
Age	≥6 years	0.90	0.26–3.09	0.87
	<6 years	Reference		
Sex	Male	1.15	0.33–3.99	0.82
	Female	Reference		
Steroid Initiation	≥7 days	2.50	0.40–15.70	0.33
	<7 days	Reference		
Antiviral use	No	0.87	0.27–2.77	0.82
	Yes	Reference		

Note: Reference groups were incomplete palsy, < 6 years of age, female sex, < 7 days to steroid initiation, and use of antiviral agents. Model diagnostics were performed: collinearity was assessed using the variance inflation factor (all < 2.0), and residual analysis did not reveal concerning deviations. * *p* < 0.05.

## Data Availability

All data and materials used and analyzed during the course of this study are available from the corresponding author upon reasonable request.

## Abbreviations

The following abbreviations are used in this manuscript:

PFP	Peripheral Facial Palsy
H–B grade	The House-Brackmann grading system
MRI	Magnetic Resonance Imaging
SD	Standard Deviation
IQR	Interquartile Range
EMG	Electromyography
CI	Confidence Interval
OR	Odds Ratio
VIF	Variance Inflation Factor
